# Deciphering molecular details of the RAC–ribosome interaction by EPR spectroscopy

**DOI:** 10.1038/s41598-021-87847-y

**Published:** 2021-04-21

**Authors:** Sandra J. Fries, Theresa S. Braun, Christoph Globisch, Christine Peter, Malte Drescher, Elke Deuerling

**Affiliations:** 1 Department of Biology, Molecular Microbiology, University of Konstanz, 78457 Konstanz, Germany; 2 Konstanz Research School Chemical Biology (KoRS-CB), University of Konstanz, 78457 Konstanz, Germany; 3 Department of Chemistry, Physical and Biophysical Chemistry, University of Konstanz, 78457 Konstanz, Germany; 4 Department of Chemistry, Computational and Theoretical Chemistry, University of Konstanz, 78457 Konstanz, Germany

**Keywords:** Structural biology, Chaperones

## Abstract

The eukaryotic ribosome-associated complex (RAC) plays a significant role in de novo protein folding. Its unique interaction with the ribosome, comprising contacts to both ribosomal subunits, suggests a RAC-mediated coordination between translation elongation and co-translational protein folding. Here, we apply electron paramagnetic resonance (EPR) spectroscopy combined with site-directed spin labeling (SDSL) to gain deeper insights into a RAC–ribosome contact affecting translational accuracy. We identified a local contact point of RAC to the ribosome. The data provide the first experimental evidence for the existence of a four-helix bundle as well as a long α-helix in full-length RAC, in solution as well as on the ribosome. Additionally, we complemented the structural picture of the region mediating this functionally important contact on the 40S ribosomal subunit. In sum, this study constitutes the first application of SDSL-EPR spectroscopy to elucidate the molecular details of the interaction between the 3.3 MDa translation machinery and a chaperone complex.

Ribosome-tethered chaperones guide the initial folding of nascent protein chains into their functional conformation^1,2^. One eukaryotic chaperone system comprises the ribosome-associated complex (RAC) as a conserved element^1^. In yeast, one of the best studied eukaryotic model systems for protein folding, RAC, is a stable heterodimer composed of the proteins Ssz1 and Zuo1 (Fig. 1b) that acts as a co-chaperone of Ssb. Stimulated by RAC, Ssb can bind a broad range of polypeptides to assist in co-translational folding^3,4^. In RAC, solely Zuo1 contacts the ribosome and thereby spans both subunits (40S and 60S) via a predicted long α-helix, referred to as middle domain (Fig. 1a,b)^5^. At the 60S subunit Zuo1’s N-terminal domain (Ssz1 binding), the J-domain (stimulation of Ssb) and the Zuo1 Homology Domain (ZHD; ribosome binding) can be found. At the small ribosomal subunit (40S) Zuo1’s C-terminus interacts with ES12, a eukaryotic expansion segment of helix 44 (H44) of the 18S rRNA (Fig. 1b). Intriguingly, the 18S rRNA reaches to the decoding center of the ribosome, which is in line with the finding that the Zuo1-ES12 contact affects translational fidelity^6^.

**Figure 1:**
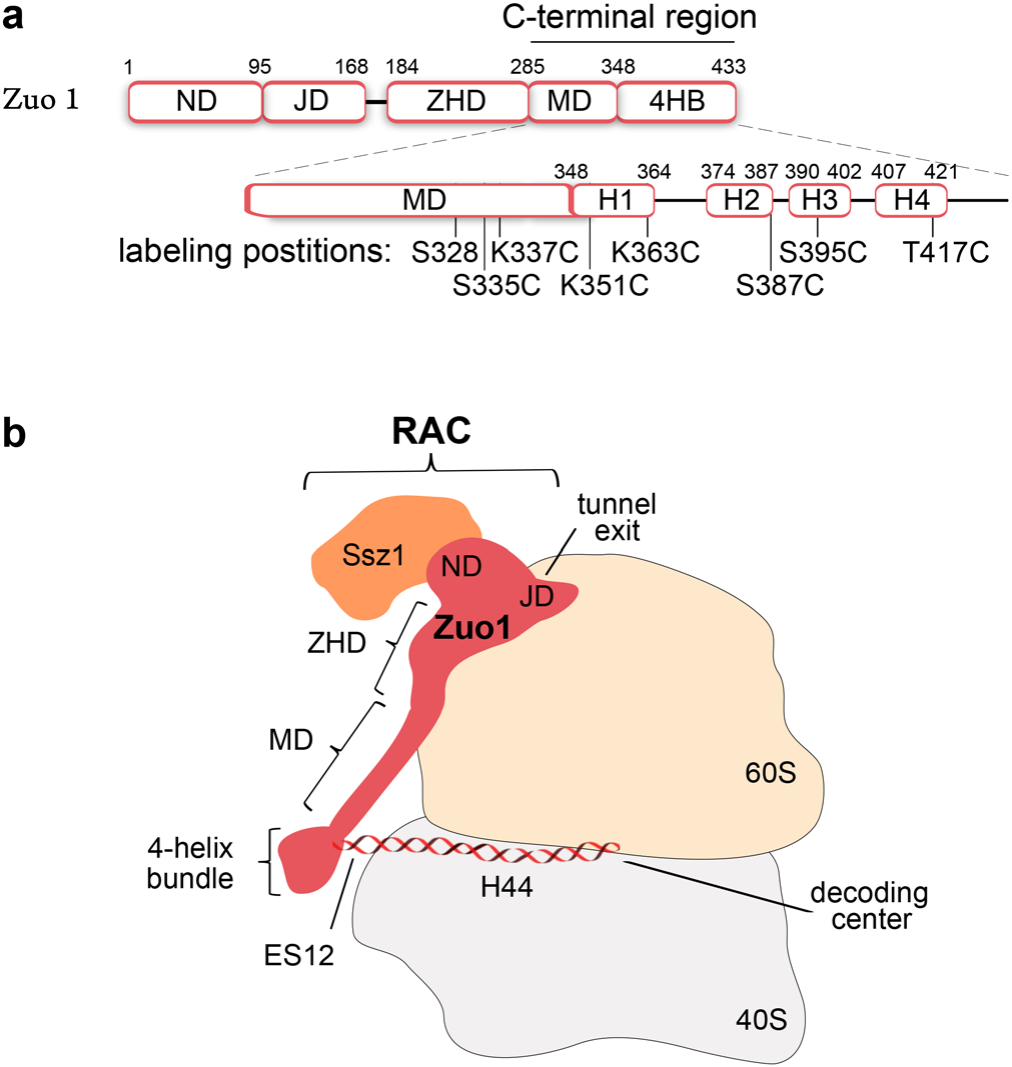
Domain organization of Zuo1 and its interaction with the ribosome. (**a**) Domains: N-terminal domain (ND), J-domain (JD), Zuo1 Homology Domain (ZHD), middle domain (MD), four-helix bundle (4HB). The labeling positions for EPR spectroscopy (cysteine substitutions) are marked. (**b**) Model of the RAC–ribosome complex.

In Zuo1, the interaction with ES12 is mediated by a positively charged patch (from aa 284 to 364) comprising the C-terminal part of the middle domain and the first helix of a C-terminal four-helix-bundle (4HB)^5–7^. A modeled α-helix as middle domain combined with the NMR structure of the 4HB^8^ were recently fitted into the cryo-EM density of the RAC–ribosome complex^5^, providing a structural model for Zuo1 C-terminal region. However, whether this model reflects the situation of full-length RAC on the ribosome is unknown. Furthermore, it is not clear how Zuo1’s C-terminal region is positioned relative to the ribosome.

To understand the molecular details of how RAC interacts with the ribosome and whether structural changes in RAC play a role in controlling this chaperone complex and its activity, it was necessary to gain insights into the conformational flexibility of RAC with and without ribosomes. In this study we applied EPR spectroscopic methods combined with site-directed spin labeling (SDSL)^9^ to analyze the functionally important Zuo1-40S contact on a molecular level. EPR spectroscopy is ideally suited to study the functional interplay between the heterodimeric RAC (107 kDa) and the ribosome (3.3 MDa^10^) as the method is not limited by size, flexibility or complexity of the system^11^. First, we analyzed local side-chain mobility to investigate in a residue-specific manner the local ribosome binding sites of Zuo1 to the 40S subunit. In addition, we provided evidence that EPR spectroscopy is well suited to report about the specific contacts between RAC and the ribosome. In a second step, we examined the structure of Zuo1’s C-terminus as well as potential conformational changes upon ribosome binding by double electron–electron resonance (DEER) distance measurements, which are suitable to reveal movements within DNA and proteins in the nanometer range^12–14^.

## Results

### Site-directed spin labeling (SDSL)

For a strategic cysteine placement, we built a working model (Fig. 1b) combining available structural fragments of Zuo1: the crystal structure of the Zuo1 homology domain (ZHD)^6^ and the NMR structure of the 4HB^8^ connected by a modeled α-helix as middle domain (Supplementary Fig. S1). An overview of all singly and doubly labelled RAC variants are listed in Table 1. Note, the RAC variants are named by their cysteines in Zuo1 e.g. RAC K351C. For EPR measurements Zuo1 was marked with the nitroxide spin label Maleimido-PROXYL^15^ (referred to as Proxyl), which was covalently attached to the sulfhydryl group of cysteines. The cysteines were introduced in the context of the authentic RAC dimer at different positions in the middle domain and the 4HB of Zuo1 (Fig. 1a).

**Table 1:** RAC variants used for EPR spectroscopy. Shown are the names of the RAC variants, their amino acid substitutions in Ssz1 and Zuo1, the location of the cysteines (labeling sites) in Zuo1’s C-terminus: middle domain or four-helix bundle (4HB) and the applied EPR spectroscopic method: continuous-wave (CW) for mobility measurements or double electron–electron resonance (DEER) for distance measurements.

Name	Substitutions	Location of Cysteines
RAC K351C	Ssz1 C81S C86S Zuo1 C167S K351C	4HB: helix 1
RAC K363C	Ssz1 C81S C86S Zuo1 C167S K363C	4HB: helix 1
RAC KR_PP K351C	Ssz1 C81S C86S Zuo1 C167S K351C K355P R395P	4HB: helix 1
RAC KR_PP K363C	Ssz1 C81S C86S Zuo1 C167S K355P R395P K363C	4HB: helix 1
RAC K351C S328C	Ssz1 C81S C86S Zuo1 C167S S328C K351C	Middle domain 4HB: helix 1
RAC K351C S335C	Ssz1 C81S C86S Zuo1 C167S S335C K351C	Middle domain 4HB: helix 1
RAC K351C K337C	Ssz1 C81S C86S Zuo1 C167S K337C K351C	Middle domain 4HB: helix 1
RAC K351C K363C	Ssz1 C81S C86S Zuo1 C167S K351C K363C	4HB: helix 1
RAC K351C S395C	Ssz1 C81S C86S Zuo1 C167S K351C S395C	4HB: helix 1 and helix 3
RAC K351C T417C	Ssz1 C81S C86S Zuo1 C167S K351C T417C	4HB: helix 1 and helix 4
RAC K363C S387C	Ssz1 C81S C86S Zuo1 C167S K363C S387C	4HB: helix 1 and helix 2
RAC K363C S395C	Ssz1 C81S C86S Zuo1 C167S K363C S395C	4HB: helix 1 and helix 3
RAC K363C T417C	Ssz1 C81S C86S Zuo1 C167S K363C T417C	4HB: helix 1 and helix 4
RAC KR_PP K351C K363C	Ssz1 C81S C86S Zuo1 C167S K351C K355P R395P K363C	4HB: helix 1

To exclude potential impacts of the introduced cysteines on the structure and function of RAC we performed multiple control experiments and simulations: The secondary structure (Supplementary Fig. S2), protein stability (Supplementary Fig. S3) and in vivo functionality (Supplementary Fig. S4) of RAC were not affected by the mutations. However, some substitutions caused a slightly weakened ribosome binding in vitro (Supplementary Fig. S5).

### Identification of a local contact point of RAC to the ribosome

**Figure 2:**
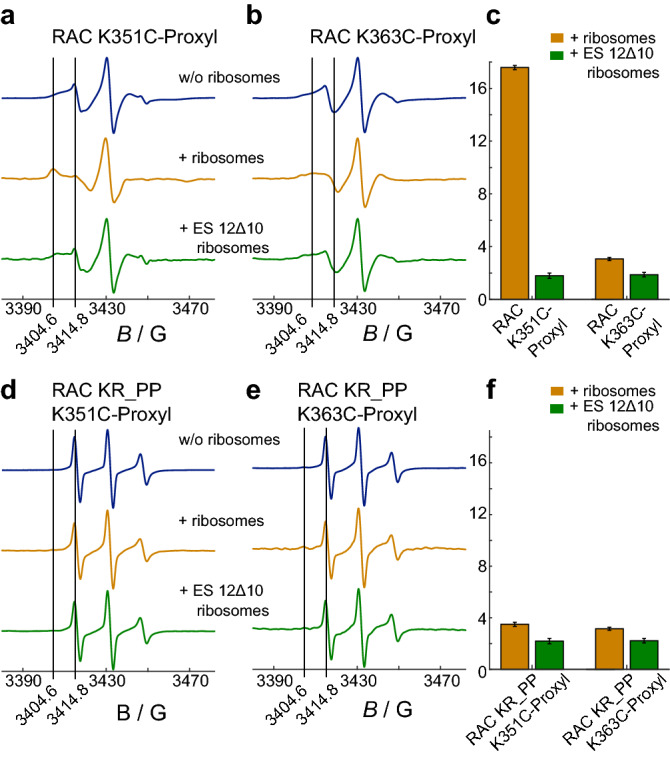
X band EPR spectra of RAC labeled in helix 1 of Zuo1’s 4HB. Shown are the spectra of (**a**) RAC K351C-Proxyl, (**b**) RAC K363C-Proxyl and (**d**,**e**) the respective variants with two prolines (KR_PP) in absence (blue) or presence of ribosomes (yellow) or ES12Δ10 ribosomes (green). Significant spectral features as represented by low field peaks at 3404.6 G and 3414.8 G are highlighted by a black line. (**c + f**), The intensity ratios at the position of the two low-field peaks (low field peak ratio, LFPR) were calculated for spectra with ribosomes and normalized to the respective ratio in absence of ribosomes. Error bars indicate the noise-low field peak signal ratio.

First, we focused on the relative orientation of Zuo1’s C-terminal region to the ribosome. Therefore, we monitored the rotational diffusion of attached spin labels via EPR spectral shape analysis. A restriction in the rotational freedom reports on local ribosome binding. Figure 2a,b shows the spectra of the Proxyl-labeled RAC variants K351C and K363C. Both residues were formerly lysines and are located in helix 1 of Zuo1’s 4HB (see Fig. 1a). The spectra could not be described by a single, isotropic spectral component, probably due to the complex environment of the label attached to the protein^16–21^. Nevertheless, in the presence of ribosomes, a slower component with a characteristic peak at 3404.6 G was found in the spectrum of RAC K351-Proxyl. The spectral change indicates that the rotational diffusion was impaired at this position (Fig. 2a). For RAC K363C-Proxyl, in contrast, only minor changes in the spectrum were observable in the presence of ribosomes (Fig. 2b). To parameterize these changes without the need of a full spectral simulation, we consulted the characteristic peak and performed a low field peak analysis by comparing the intensities at two distinct points in the low magnetic field region (low field peak ratio = LFPR; see “Methods” section). The ratios were normalized to the respective spectra without ribosomes (Fig. 2c).

To show that the strong effect of RAC K351C-Proxyl is specific and derived from ribosome binding we applied two different strategies to prevent the interaction. In the first approach we used ribosomes lacking 10 terminal bases in ES12^6^ (referred to as ES12∆10), which drastically reduced the affinity of RAC to these ribosomes (Supplementary Fig. S5b). The weakened interaction of RAC K351C-Proxyl to ES12∆10 ribosomes is reflected in the spectral shape (Fig. 2a, green spectrum) and a reduced LFPR (Fig. 2c). Since the spectrum of RAC K363C-Proxyl is less affected by ribosome binding in general (Fig. 2b, yellow spectrum), the effect of ES12∆10 ribosomes is also smaller compared to the K351C-Proxyl variant (Fig. 2a–c).

In the second approach, we disrupted the RAC–ribosome interaction by a proline-induced unfolding of the 4HB. The replacement of a lysine and an arginine in helix 1 by prolines (KR_PP) resulted in a loss of the secondary structure, which was predicted by molecular dynamic simulations and verified by DEER (Supplementary Fig. S8). The single cysteine proline variants (RAC KR_PP K351C/K363C) showed a strongly reduced affinity to ribosomes (Supplementary Fig. S5a), which was completely lost in presence of ES12∆10 ribosomes (Supplementary Fig. S5b). Hampered ribosome binding became also visible in the CW spectra and LFPR: the spectral shape of RAC KR_PP K351C-Proxyl and RAC KR_PP K363C-Proxyl is not affected by the presence of wt or ES12∆10 ribosomes (Fig. 2d–f). The slightly increased LFPR for samples with wt ribosomes can be explained by the residual binding affinity (Supplementary Fig. S5a).

Taken together, both controls interfering with the 40S-Zuo1 contact showed that the slower rotational mobility (and high LFPR) for RAC K351C-Proxyl + 80S ribosomes can be assigned to local ribosome binding at this position.

### Existence of a four-helix bundle and a long α-helix

Next we analyzed the conformation of Zuo1’s C-terminal region with and without ribosomes. We aimed at a series of long-range distance restraints between the middle domain and the 4HB as well as within the helix bundle. For each distance restraint, one spin label was placed in helix 1 of the 4HB (K351C or K363C) and a second label in the middle domain or in helix 2–4. We performed Q-band double electron electron resonance (DEER) experiments to obtain experimental distance restraints between the labels. Expected distance distributions were calculated on the basis of our structural working model with the open-source software MMM^22^ (Supplementary Fig. S1, see “Methods”).

**Figure 3:**
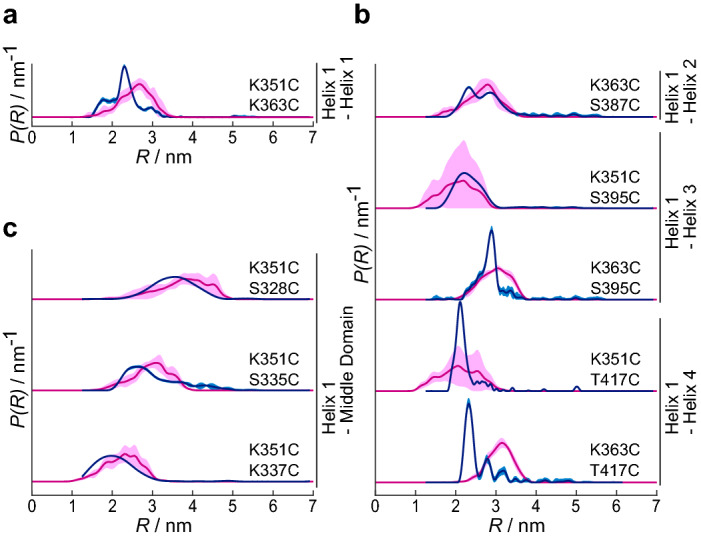
Experimental distance distributions *P*(*R*) of RAC w/o ribosomes (blue lines) compared to simulated distance restraints based on our working model (magenta lines). The data were obtained by background correction with the use of DEERNet^12^ and Tikhoniv regularization. Shaded areas represent the uncertainty range according to the validation (light blue, see “Methods”) or standard deviation of clustered structures from two independent simulations (light magenta). RAC was labeled at (**a**) two sites within helix 1 of Zuo1’s 4HB, (**b**) one site in helix 1 and a second site in helix 2–4, or (**c**) one site in helix 1 and a second site in the middle domain.

**Figure 4:**
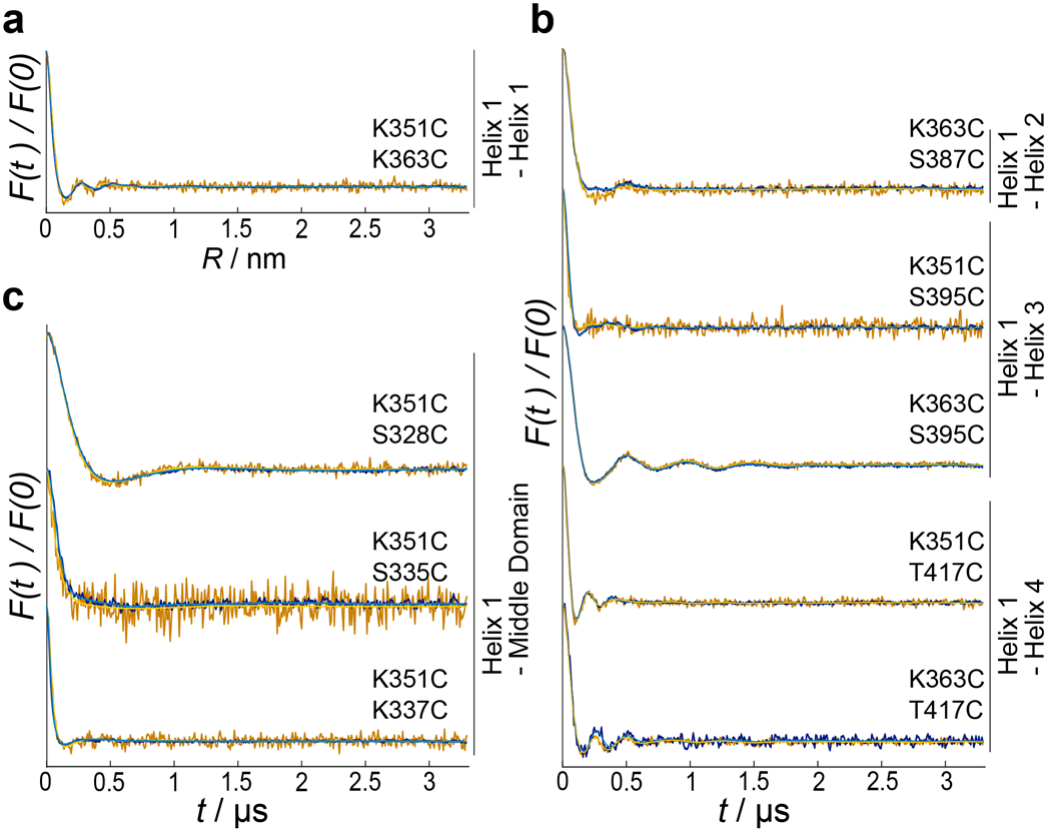
DEER form factors do not differ for RAC with and w/o ribosomes. Similar experimental form factors *F*(*t*) = *V*(*t*)/*B*(*t*) light colors) and corresponding Tikhonov regularization fits (dark colors) of Proxyl-labeled RAC variants in solution (blue) or complexed with ribosomes (yellow) indicate that RAC preserves its conformation upon ribosome binding. RAC was labeled at (**a**) two sites within helix 1 of Zuo1’s 4HB, (**b**) one site in helix 1 and a second site in helix 2–4, or (**c**) one site in helix 1 and a second site in the middle domain. Related DEER raw data is shown in Supplementary Fig. S6.

The measured distance distributions of RAC in solution (blue lines) and the respective predictions based on the working structural model (magenta lines) are shown in Fig. 3. In most cases, the experimentally determined distances match the predictions (magenta lines) based on the working structural model or are at least in a similar range. To see whether conformational rearrangements occur upon ribosome binding, we conducted the same set of measurements with ribosome-associated RAC. Interestingly, the DEER form factors do not significantly change in the presence of ribosomes (Fig. 4), suggesting that the RAC-conformation is astonishingly rigid in solution and does not significantly change upon ribosome binding. The data presented up to this point were conducted with empty 80S ribosomes (i.e. without a nascent polypeptide) but as the binding mode of RAC may differ during translation, we repeated two measurements with ribosomes in the translational state (see “Methods” section). Interestingly, the obtained form factors are comparable to those with empty ribosomes (Supplementary Fig. S7).

Collectively, our DEER distance measurements are consistent with the hypothesis that the 4HB and alpha-helical conformation for the middle domain exist in the context of full-length RAC. Surprisingly, this conformation of Zuo1 C-terminus seems neither influenced by ribosome binding nor by the translation state of the ribosomes.

## Discussion

The functional interplay between RAC and the ribosome is not only essential for proper co-translational folding but also for high translational fidelity^6,23^.

Earlier cryo-EM analysis^5^ revealed that yeast RAC contacts both subunits of the 80S ribosome using three binding sites in Zuo1 (contact C1–C3), with C1 and C2 binding to the 60S subunit close to the polypeptide exit tunnel and C3 in the C-terminus of Zuotin contacting a helical RNA element (ES12) of the 40S subunit that elongates into the decoding center of the ribosome (Fig. 1B). This finding raises the attractive hypothesis that polypeptides with folding problems may recruit RAC to regulate their elongation speed in order to recruit Ssb and allow the RAC-Ssb chaperone system to assist their folding. Especially the contact between Zuo1’s C-terminus and the ribosomal extension segment ES12 may influence translational accuracy and speed. To learn more about the conformation and positioning of Zuo1’s C-terminus we employed EPR spectroscopy. Distance restraints obtained by DEER allowed us to complement the current structural model of Zuo1’s C-terminal region. Our working model was composed of the atomic structure of the C-terminal four-helix bundle (4HB) and a modeled long α-helix as middle domain^5,6^. The sequence of the middle domain predicts an α-helix, but the secondary structure has not been experimentally determined thus far as the crystallization of this region is challenging^24^. Here, we provided the first experimental evidence that the 4HB and the long α-helix of middle domain exist in full-length RAC, both in solution and on the ribosome.

Unexpectedly, we found that the conformation of Zuo1’s C-terminus remains unchanged upon binding to vacant and nascent chain-carrying ribosomes. There were at least no detectable changes above the lower DEER detection limit of 1.8 nm^13^. The observed rigidity of the entire C-terminal region implies that a conformational switch for the assumed RAC-mediated signal transduction between the decoding center and the nascent polypeptide exit tunnel of the ribosome might take place in another region of Zuo1.

Knowing the structural conformation of Zuo1’s C-terminal region, we also conducted CW mobility measurements to position Zuo1 relative to the 40S ribosomal subunit.

**Figure 5:**
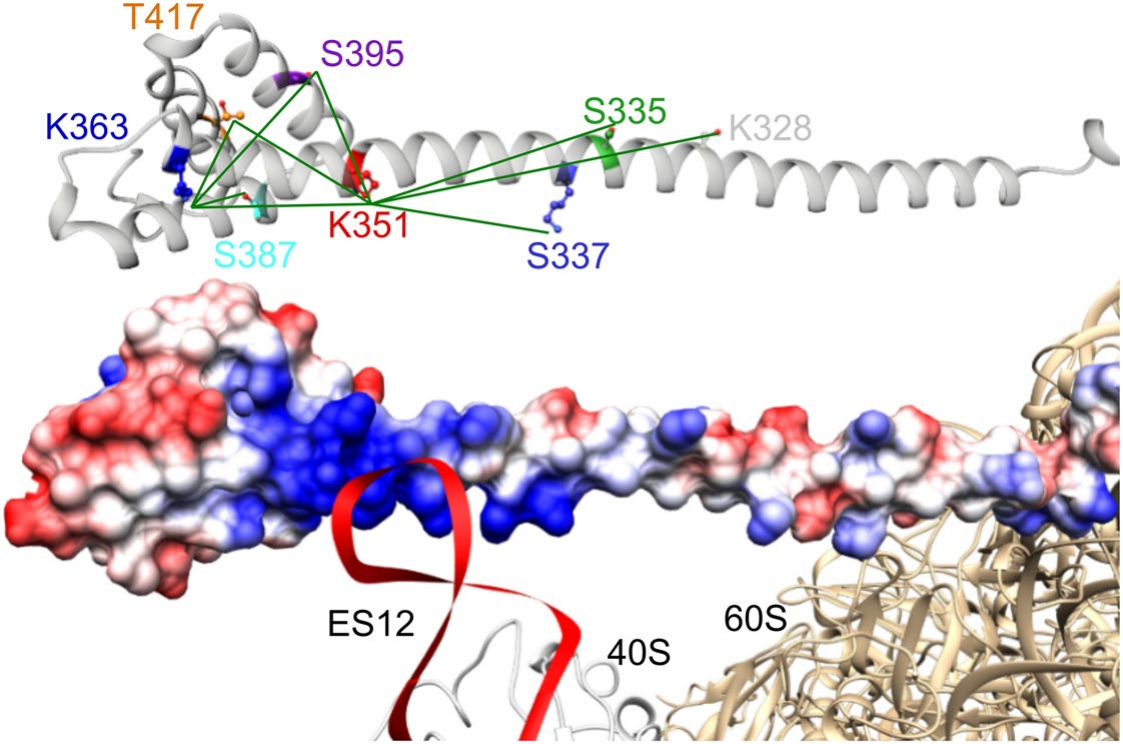
Structural model of Zuo1’s C-terminal region and its interaction with ES12 involving K351. The upper panel shows the modelled middle domain and four-helix bundle of Zuo’s C-terminus in a ribbon presentation (homology model based on PDB 2LWX and 5DJE). Residues used for EPR measurements are shown as colored sticks. Measured distance distributions are indicated by green lines. The lower panel shows the model of Zuo1 C-terminus in a space filling illustration (blue: electropositive surface areas and red electronegative surface area) and its putative interaction with ES12 (red) of the 40 subunit (beige) involving amino acid side chain K351 of Zuo1. The homology model of 4HB is placed with respect to the ribosome structure^25^ (PDB 3J78) in the orientation described by Lee et al.^6^ (for detail see “Methods” section).

The knowledge that K351 but not K363 is close to ES12, finally allowed us to position the C-terminal region of Zuo1 relative to the ribosome. We rotated our structural model in a way that K351 is close to ES12, as illustrated in Fig. 5.

In summary, this study shows for the first time molecular details of the interaction of RAC with the 40S subunit of the ribosome. Moreover, the EPR techniques applied herein reflect an important experimental advance to decipher the dynamic interaction of this chaperones with the ribosome. Now the way is paved to understand the mechanistic details of the interactions of RAC with ribosomes which should allow to decipher its modus operandi on translating ribosome.

## Methods

### Strains, plasmids and growth conditions

Yeast strains used in this study are BY4741 (MATa; his3Δ1; leu2Δ0; met15Δ0; ura3Δ0) and BY4741 ssz1∆, zuo1∆ (RAC∆). ES12∆10 80S ribosomes were purified from strain KAY488, which lacks ~ 150 chromosomal rDNA repeats and expresses the plasmid pNOY373 ES12∆10^26^. Plasmids are listed in Supplementary Table S1. For the construction of the respective RAC variants, first, the three endogenous cysteines (Ssz1 C81 C86; Zuo1 C167) were substituted by serines, followed by the introduction of cysteines at the desired amino acid positions. Replacement and introduction of cysteine codons in SSZ1 and ZUO1 were performed according to the guidelines of the Q5^®^ Site-Directed Mutagenesis Kit (NEB) or by a Fusion-PCR strategy introducing the mutation site via overlapping primers.

Unless otherwise indicated, yeast cells were grown in rich medium (YPD) or defined synthetic complete (SC) media (6.7 g/L Bacto-Yeast Nitrogen Base w/o amino acids, 2 g amino acid mix, 2% (w/v) dextrose) at 30 °C. For growth analysis, BY4741 or RAC∆ cells were transformed with yeast plasmids (Supplementary Table S1) Exponentially growing cultures of transformants were diluted to OD_600_ = 0.4, spotted in fivefold serial dilutions on SC plates w/o leucin and uracil and incubated at 30 °C for 2 or 3 days. Plates supplemented with translation inhibitory drugs contained 25 μg/mL hygromycin B or 0.75 μg/mL l-Canavanin (medium w/o arginine), respectively.

### Protein expression and purification

For recombinant expression and purification of RAC *Escherichia Coli* BL21 (DE3)*/pRARE cells were transformed with a variant of *His6-SUMO-SSZ1-ZUO1* (Table 1 and Supplementary Table S1). Cells were grown in liquid culture to an OD_600_ = 0.6 at 30 °C and expression of RAC was induced with 0.5 mM IPTG for 3 h at 30 °C. Cells were harvested, resuspended in lysis buffer (40 mM HEPES pH 7.4, 1 M KAc, 5% (v/v) glycerol, 2 mM β-Mercaptoethanol, 1 mM PMSF, 1 mM EDTA, protease inhibitors, DNaseI) and lysed by French Press. Lysates were cleared by centrifugation (18,000 xg, 30 min), supernatant treated with 10 mM MgCl_2_ to complex residual EDTA and finally applied to Ni-IDA resin (Protino; Macherey–Nagel) for 30 min at 4 °C. The matrix was alternately washed twice with low salt buffer (40 mM HEPES pH 7.4, 100 mM KAc, 5 mM MgCl_2_, 5% (v/v) glycerol, 2 mM β-Mercaptoethanol), high salt buffer (like low salt with 1 M KAc) and low salt buffer respectively. Finally, the His6-SUMO-Ssz1–Zuo1 complex was eluted in elution buffer (low salt buffer with 300 mM imidazole). Elution fractions were mixed with Ulp1 protease (5 μg/mg total protein) to remove His6-SUMO and dialyzed against RAC buffer (low salt buffer without β-Mercaptoethanol) for 3 h. As second purification step RAC was applied to an anion exchange column (ResourceQ, GE Healthcare). All chromatogram peaks were analyzed by SDS-PAGE and RAC containing fractions were pooled and dialyzed against RAC buffer. In a last purification step RAC was purified by size exclusion chromatography through a HiLoad Superdex 200 (GE Healthcare) column equilibrated with RAC buffer. Purity was verified by SDS-PAGE and fractions containing highly pure RAC were pooled, flash frozen and stored at − 80 °C.

To test stability of purified RAC variants, protein samples were incubated for 0 h and 16 h at room temperature and subsequently analyzed in Coomassie Blue-stained SDS-PAGE (2 µg per sample loaded).

### Purification of 80S ribosomes

Yeast 80S ribosomes were either purified from BY4741 or KAY488 + pNOY373 ES12∆10. To obtain ribosomes free of endogenous RAC the protocol includes a mild salt-wash step with 150 mM KCl. 12 L culture were grown to OD_600_ = 0.8, harvested and pellets flash frozen in liquid nitrogen. Cells were opened in a pre-cooled Retch Mill MM400 (30 Hz for 60 s) and powder was resuspended in Lysis Buffer (50 mM HEPES pH 7.4, 300 mM NaCl, 6 mM MgCl_2_, 0.5% (v/v) Nonidet-P40, 2 mM DTT, 1 × cOmplete protease inhibitor cocktail (Roche), 1 mM PMSF). Cellular debris was removed by centrifugation (16,000×*g*, 30 min, 4 °C), lysates applied to a 60% (w/v) sucrose cushion [50 mM HEPES pH 7.4, 50 mM KCl, 10 mM MgCl_2_, 5 mM EDTA, 0.5 × cOmplete protease inhibitor cocktail (Roche)] and centrifuged for 20 h at 184,000×*g* (45,000 rpm) at 4 °C in a Ti50.2 rotor (Beckmann coulter). Ribosomal pellets were resuspended in resuspension buffer (50 mM HEPES pH 7.4, 150 mM KCl, 6 mM MgCl_2_, 1 mM DTT, 6.8% (w/v) sucrose) by shaking (150 rpm) on ice for 3 h. The crude ribosomal extract was then treated with 1 mM puromycin (InvivoGen) for 30 min at RT to release nascent chains and afterwards centrifuged for 20 min at 20,000×*g* at 4 °C. The ribosomal subunits were separated on 15–45% (w/v) sucrose gradients (50 mM HEPES pH 7.4, 150 mM KCl, 2 mM MgCl_2_, 1 mM DTT), centrifuged at 17,500 rpm and 4 °C for 17 h (SW28-rotor, Beckmann Coulter). 80S containing fractions were collected and buffer exchanged to RAC buffer (40 mM HEPES pH 7.4, 100 mM KAc, 5 mM MgCl_2_) in 100,000 kDa MWCO centrifugal filters (Amicon Ultra-4, Millipore). Aliquots of 20 µL ribosomes were flash frozen and stored at − 80 °C.

To purify ribosomes that may still carry the nascent chain we adapted the protocol as follows: Faster harvest by vacuum filtration, no treatment with puromycin and collection of only the polysome fraction (translating ribosomes).

### In vitro ribosome binding assays

To test the ribosome binding ability of the RAC variants, 1 µg 80S ribosomes (wt or ES12∆10) were mixed with 0.8 µg RAC and incubated for 30 min at 30 °C to allow the formation of RAC–ribosome complexes. Unbound RAC was removed via centrifugation through a 25% (w/v) sucrose cushion at 200,000×*g* (S140-AT rotor) for 90 min. The ribosome-containing pellet was resuspended in RAC buffer (40 mM HEPES pH 7.4, 100 mM KAc, 5 mM MgCl_2_). Samples were separated by SDS-PAGE, wet blotted on nitrocellulose membranes (GE Healthcare) and stained by Ponceau S. The stained membrane was used for quantification. The signal intensity of RAC (Szz1 + Zuo1 signal) was determined by Fiji and divided by the signal intensity of the ribosomal protein band below RAC (internal loading reference). For background correction the respective ratio of the ribosome sample (80S ribosomes w/o RAC) was subtracted. The ratio for wt RAC bound to wt 80S ribosomes was set to 100%. Error bars represent standard error of the mean (s.e.m) of at least three independent experiments.

Zuo1 and uL22 (Rpl17A) were detected by immuno-staining with polyclonal antibodies^27^. Primary antibodies were detected by HRP-coupled secondary antibodies (711-035-152, Dianova) and visualized with the Fusion SL (PEQLAB) imaging system.

### Sample preparation for EPR spectroscopy

For EPR spectroscopy purified RAC variants carrying a single or two cysteines were labeled with the nitroxide spin label Maleimido-PROXYL^15^ (3-Maleimido-2,2,5,5-tetramethyl-1-pyrrolidinyloxy, Sigma-Aldrich). Prior labeling, cysteines were reduced with a six-fold molar excess of DTT over SH-groups for 30 min at 4 °C. DTT was removed by a desalting column (Zeba spin, Thermo Fisher), followed by the immediate addition of a six-fold molar excess of spin label. The samples were labeled for 2 h or overnight at 4 °C. Unbound label was removed in two consecutive desalting steps via desalting columns (Zeba spin, Thermo Fisher), equilibrated with RAC buffer (40 mM HEPES pH 7.4, 100 mM KAc, 5 mM MgCl_2_). Samples were concentrated to 50–190 µM using centrifugal filters (Amicon Ultra, MWCO 30 kDa, Millipore). To obtain RAC–ribosome complexes, labeled RAC was incubated with a 20% molar excess of purified yeast 80S ribosomes for 30 min at 30 °C.

### X-band continuous wave (CW) EPR spectroscopy

CW EPR spectroscopy was performed with singly labeled RAC at X-band (9.645 GHz) frequency in aqueous solution (RAC buffer: 40 mM HEPES pH 7.4, 100 mM KAc, 5 mM MgCl_2_) at room temperature. Measurement parameters were adjusted such that the spectral line shape was not distorted by overmodulation or saturation effects. Typical settings on the used EMXnano benchtop spectrometer (Bruker Biospin) were a power of 3.162 mW, a modulation amplitude of 0.8 G at a modulation frequency of 100 kHz. Samples were loaded into capillary pipettes ringcaps with 1 mm inner diameter (Hirschmann), and sealed with Hemato-Seal capillary tube sealant (Fisherbrand). The magnetic field-axis was recalculated to the microwave-frequency of 9.6355 GHz, and the spectra were normalized to the maximum amplitude of the center field peak. The intensity low field peak ratio (LFPR) = *I* (3415.3 G)/*I* (3404.6 G) was calculated. LFPR were normalized to the LFPR of the protein spectrum in solution for each RAC variant. Error bars indicate the noise-low field peak ratio. Since concentration and thus signal-to-noise ratios were worse for spectra in presence of ribosomes, they were Savitzky–Golay filtered with an order of 2 and a frame of 101 for better illustration.

### Q-band double electron–electron resonance (DEER) spectroscopy

For DEER experiments samples (with 20% (v/v) glycerol-d8) were loaded into quartz tubes (Fused quartz tubing, Technical Glass Products; 2 mm inner diameter) and shock frozen in liquid nitrogen before measurement. The experiments were performed using an ELEXSYS E580 spectrometer equipped with a Q-band resonator (ER5106QT-2, Bruker Biospin) and a 150 W traveling-wave tube (TWT) amplifier (Applied Systems Engineering, Fort Worth, USA). Samples were held on cryogenic temperatures (50 K) with the EPR Flexline helium recirculation system (CE-FLEX-4K-0110, Bruker Biospin, ColdEdge Technologies) comprising a cold head (expander, SRDK-408D2) and a F-70H compressor (both SHI cryogenics, Tokyo, Japan), controlled by an Oxford Instruments Mercury ITC.

The DEER experiment was performed using a four-pulse sequence, using rectangular pulses (π/2_obs_ − τ_1_ − π_obs_ − t′ − π_pump_ − (τ_1_ + τ_2_ − t′) − π_obs_ − τ_2_ − Echo). The integrated echo amplitude was recorded as a function of the dipolar evolutions time t′. The pump and observer pulses were positioned on the global spectral maximum and close to the local maximum (shifted by 70 MHz), respectively. The pump and observer pulse length were adjusted for each sample individually to obtain a flip angle of π for the pump and π/2 and π for the observer pulses. The pulse separation time τ1 was 400 ns and the dipolar evolution time τ2 was 4000 ns. Typical pump pulses were in the range of 13 and 24 ns, observer pulses were 24–35 ns. An eight-step phase cycle was used; proton modulations were suppressed by adding 8 DEER time-traces for different τ_1_ values with a τ_1_ increment of 16 ns A complete DEER experiment was performed as a 2D experiment, where one dimension was the time axis and the second dimension the axis of individual scans. The scans were subjected to phase correction individually and subsequently summarized.

DEER data sets were analyzed using the DeerAnalysis 2018 software package for MATLAB^28^. Extraction of the dipolar evolution function was achieved by background correction with the generic four network ensemble DEERNet^29^ followed by model-free Tikhonov regularization. The optimum regularization parameter α was determined using the L-curve corner criterion^30^. Tikhonov validation was performed with an ensemble of reconstructed background models from DEERNet combined with 5 noise realizations each with the help of the automated validation tool of the DeerAnalysis 2018 software^29^. Form factors were normalized to the modulation depth. All resulting distance distributions *P*(*r*) were normalized such that ${\int }_{R}P(R)$ = 1. Shaded areas in Figs. 3 and Supplementary Fig. S7 represent uncertainties of the distance distributions derived from data post-processing using an ensemble of reconstructed background models from DEERNet.

### Molecular dynamics simulations

#### Model generation

The structural models of the 4HB mutants are based on the NMR structure^8^ (PDB 2LWX). For the extended model of 4HB including the middle domain of Zuo1 the helical part was extended with the comparative modeling software MODELER^31^ by combining an ideal helix for the middle domain extension with the NMR structure of 4HB as templates for the final model. To introduce the cysteine and proline point mutations we used chimera, where the most probable side chain rotamer of cysteine is replacing the previous amino acid.

For a strategic cysteine placement we extended the model further with MODELLER by the crystal structure of the Zuo1 homology domain (ZHD)^32^ (PDB 5DJE) and placed all 4HB-MD and ZHD with respect to the ribosome structure^25^ (PDB 3J78) in the orientation described by Lee et al.^6^.

#### Molecular dynamics

All molecular dynamics simulations were obtained by GROMACS version 2016.3^33,34^ using the CHARMM36m^35,36^ force field together with the tip3p^37^ water model. CHARMM-GUI^32,38,39^ was used to prepare the starting structures. The simulation box was set to dodecahedron shape and defined in such a way that the minimum distance of the structure and the box was at least 1.5 nm. Subsequently the protein was solvated with water and neutralized by sodium chloride. Two independent simulations have been performed for each structural model.

Following simulation settings have been applied. The Leap frog integrator was utilized together with all bonds being constrained by the LINCS algorithm^40^ in order to enable a time-step of 2 fs. Long range coulomb interactions were calculated by particle mesh Ewald (PME)^41^ method with a cutoff of 1.2 nm. A modified cutoff scheme for short-ranged electrostatic and Lenard Jones interactions of 1.2 nm, where a switching function is applied to smoothly approach the cutoff between 1.0 and 1.2 nm, was used. The neighbor list was updated every 20 steps. Initially all systems were energy minimized with steepest-descent algorithm for 5000 steps. In the next step an equilibration simulation followed (25 ps) in a canonical (NVT) ensemble was carried out where heavy atoms have been position restrained. The actual production simulations (1000 ns) were carried out in an isobaric-isothermal (NPT) ensemble without position restraints. The temperature was maintained at 298 K by the Nose–Hoover^42,43^ algorithm with a period of the temperature fluctuations at equilibrium set to 1 ps. Constant pressure was maintained at 1 atm using isotropic Parrinello–Rahman pressure coupling^44^ with a pressure relaxation time of 5 ps.

#### Cluster analysis and MMM calculations

In order to get representative structures for DEER spectra simulations with the MMM^22^ package, a cluster analysis was performed for the simulations of the structural models. We used the gromos clustering method^45^ with a cutoff of 0.15 nm for the root mean square deviation (RMSD) of the α-carbon atoms in the case of the 4HB model and 0.2 nm for the proline mutant and the extended 4HB-middle domain model. For every simulation, the cluster analysis was based on 1000 frames. The obtained cluster centers from clusters with more than 50 members have been subsequently processed with MMM to simulate the DEER distance distributions. In the next step, the calculated DEER distance distributions have been averaged for every structural model with weights corresponding to the cluster size and standard deviation. The standard deviation. The lower boundary was set to 0.

## Supplementary Information

Supplementary Information.

## Data Availability

Data referring to Figs. 2, 3 and 4 and Supplementary Figs. S6–8, and raw data of EPR measurements and molecular dynamics simulations that support the findings of this study have been deposited in “zenodo” with the accession codes “md5:cae27d869e5bfb1c73f50956d3c37f45”, “md5:8693766997b05f74541ceaa0db204a68”, “md5:9dbd95b91df9c4ca400755b194cf48a2”, and “md5:b0a192a4f388a0cd4448c96f43b8bbf0” (https://zenodo.org/record/4460471#.YA3ghU-g_jk).
