# Supplementary material for: Deciphering molecular details of the RAC–ribosome interaction by EPR spectroscopy
Source: Sci Rep. 2021 Apr 21;11:8681. doi: 10.1038/s41598-021-87847-y (PMC8060413; doi:10.1038/s41598-021-87847-y)
Supplement: Supplementary file 1 — Supplementary Information. [file 41598_2021_87847_MOESM1_ESM.pdf]

Supporting information for "Deciphering molecular details of the RAC-ribosome interaction by EPR spectroscopy"

Sandra J. Fries[1,2‡], Theresa S. Braun[2,3‡], Christoph Globisch[4], Christine Peter[4], Malte Drescher[3]* and Elke Deuerling[1]*

[1] Department of Biology, Molecular Microbiology, University of Konstanz, 78457 Konstanz, Germany

[2] Konstanz Research School Chemical Biology (KoRS-CB), University of Konstanz, 78457 Konstanz, Germany

[3] Department of Chemistry, Physical and Biophysical Chemistry, University of Konstanz, 78457 Konstanz, Germany

[4] Department of Chemistry, Computational and Theoretical Chemistry, University of Konstanz, 78457 Konstanz, Germany

‡These authors contributed equally

* Corresponding authors: malte.drescher@uni-konstanz.de, elke.deuerling@uni-konstanz.de

A. METHODS S2

Plasmids used for protein expression (Table S1) S2

B. CONTROL EXPERIMENTS & SUPPLEMENTARY FIGURES S3

Ribbon representation of the 80S ribosome with parts of Zuo1 (Figure S1) S3

Stability of the structural models (Figure S2) S4

Protein stability of purified RAC variants (Figure S3) S5

In vivo functionality test (Figure S4) S6

In vitro ribosome binding ability of RAC variants (Figure S5) S6

Form factors of RAC with vacant ribosomes (Figure S7) S9

Proline-induced unfolding (Figure S8) S10

C. REFERENCES S11

A. METHODS

Plasmids used for protein expression (Table S1)

Table S1 Plasmids used in this study

Plasmid	Description	Reference
pNOY373 ES12Δ10	*LEU2* yeast 2 micron plasmid carrying a single copy of rDNA repeat under control of its native promoter; 10 bases deleted in ES12 (18S rRNA)	[1]
pRS315	*LEU2* yeast centromeric vector	[2]
pRS316	*URA3* yeast centromeric vector	[2]
pRS315 Ssz1	pRS315 carrying *SSZ1* under control of its native promoter	This study
pRS315 Ssz1 C81S C86S	pRS315 Ssz1 with C81 and C86 substituted to serine	This study
pRS316 Zuo1	pRS316 carrying *ZUO1* under control of its native promoter	[3]
pRS316 Zuo1 C167S	pRS316 Zuo1 with C167 substituted to serine; basic plasmid for respective cysteine variants of Zuo1 (see Table 1)	This study
pSUMO Ssz1 C81S C86S Zuo1 C167S	*E. coli* expression plasmid with SUMO-His6-Ssz1 C81S C86S Zuo1 C167S construct under control of T7-promoter; basic plasmid for respective cysteine variants of Zuo1 (see Table 1)	This study

B. CONTROL EXPERIMENTS & SUPPLEMENTARY FIGURES

Ribbon representation of the 80S ribosome with parts of Zuo1 (Figure S1)

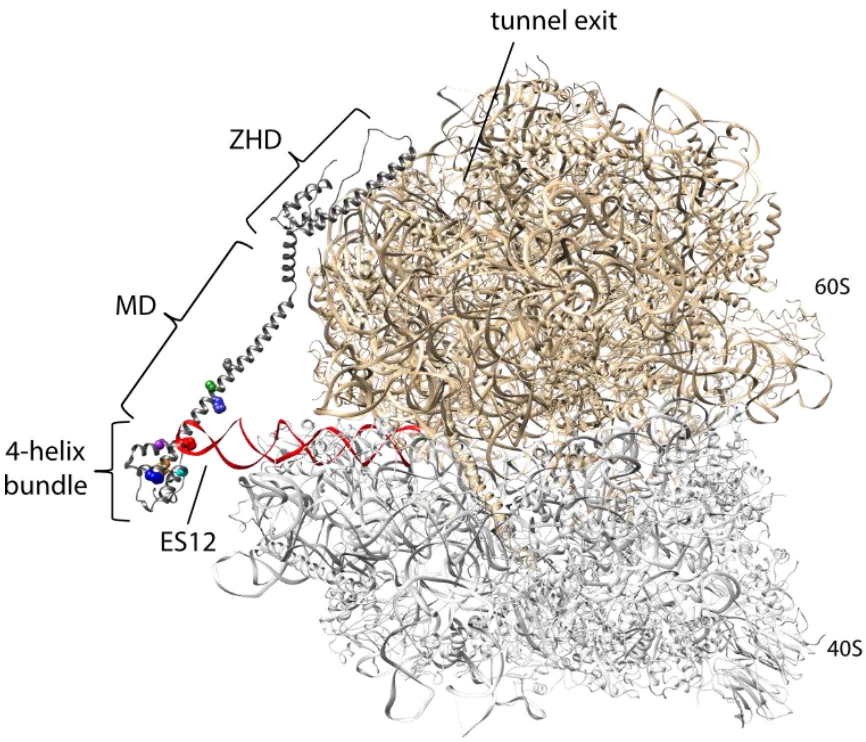

Fig. S1 Ribbon representation of the yeast 80S ribosome[4] (PDB 3J78) with parts of Zuo1. The crystal structure of the ZHD[1] (PDB 5DJE) and the NMR structure[5] (PDB 2LWX) of the 4HB are connected by a modeled long alpha-helix as MD and assigned to the ribosome based on current knowledge[1]. Labeling positions are depicted in different colors.

Stability of the structural models (Figure S2)

To check whether the cysteine mutations affect the secondary structure of Zuo1's C-terminus we performed molecular dynamics simulations with either a structural model comprising all cysteines in the 4HB (Fig. S2a) or all cysteines in the middle domain and helix 1 of the 4HB (Fig. S2b). In both cases the structures remained stable in two independent simulations over 1000 ns, indicating that even the combination of more than two introduced cysteines is unlikely to interfere with the protein structure.

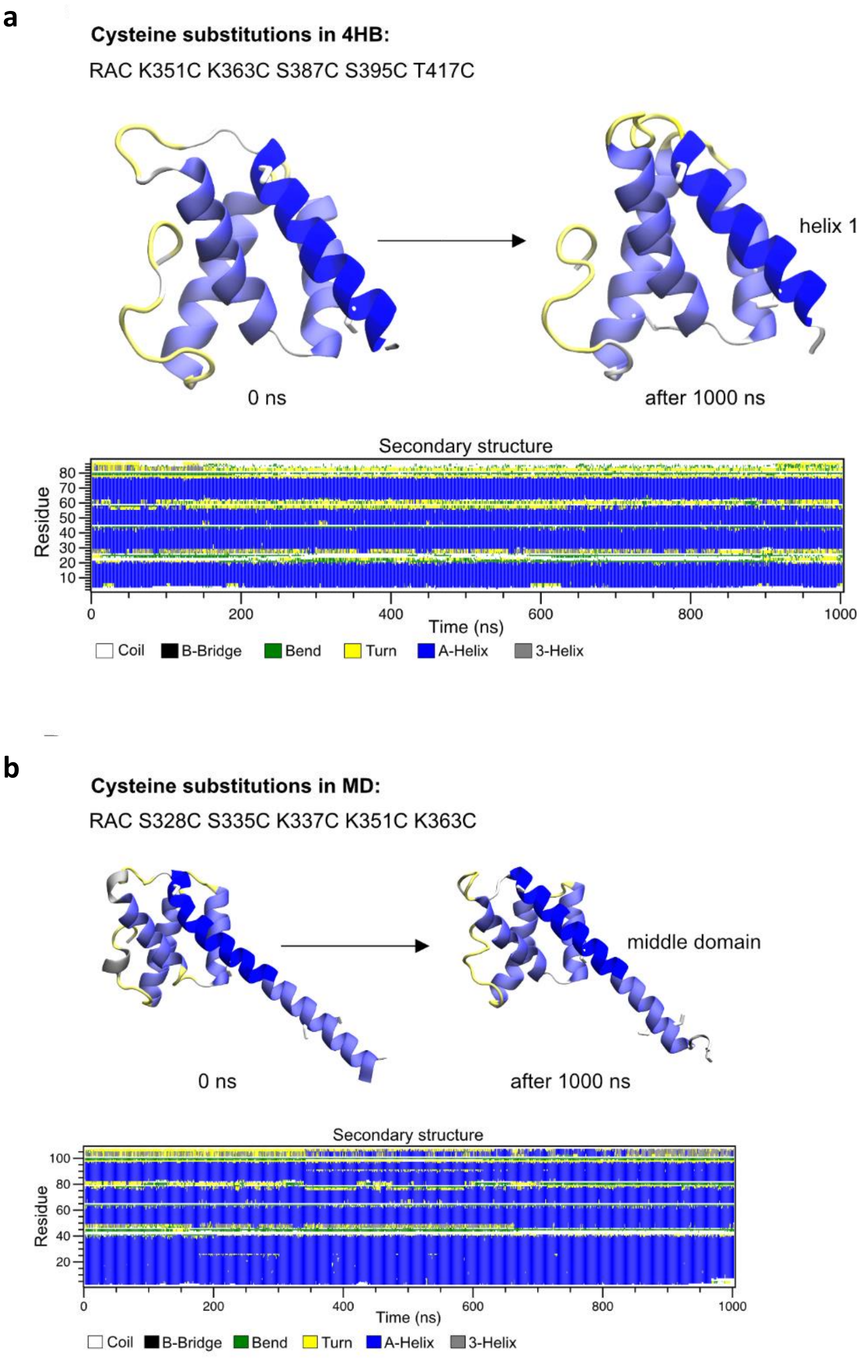

Fig. S2 Stability of the structural models combining either all cysteine in the 4HB (**a**) or all cysteine's in the MD + helix 1 of 4HB (**b**) in molecular dynamics simulations. Cartoon representation of the starting and end structures of the simulation with depicted cysteine mutations. The lower panels show the timeline of the secondary structure of the proteins. The conservation of the secondary structure elements, especially the helical parts, together with the side-to-side representation of the starting and final conformations nicely demonstrate the stability of the structural models over the simulation time. Both models behave similarly in a second independent simulation (data not shown).

Protein stability of purified RAC variants (Figure S3)

To assure that RAC remains stable in the timescale of measurements, we performed a stability test with the purified protein complexes (Fig. S3). As the CW mobility measurements were conducted at room temperature for several hours, we compared the protein pattern of purified RAC directly after thawing and after 16 h at RT. There was no visible degradation for the single cysteine variants applied to CW EPR spectroscopy and only very slight degradation in two double cysteine variants (RAC K351C S95C and RAC K351C K337C). The mild degradation of these cysteine variants was not critical since DEER experiments were recorded in a frozen state.

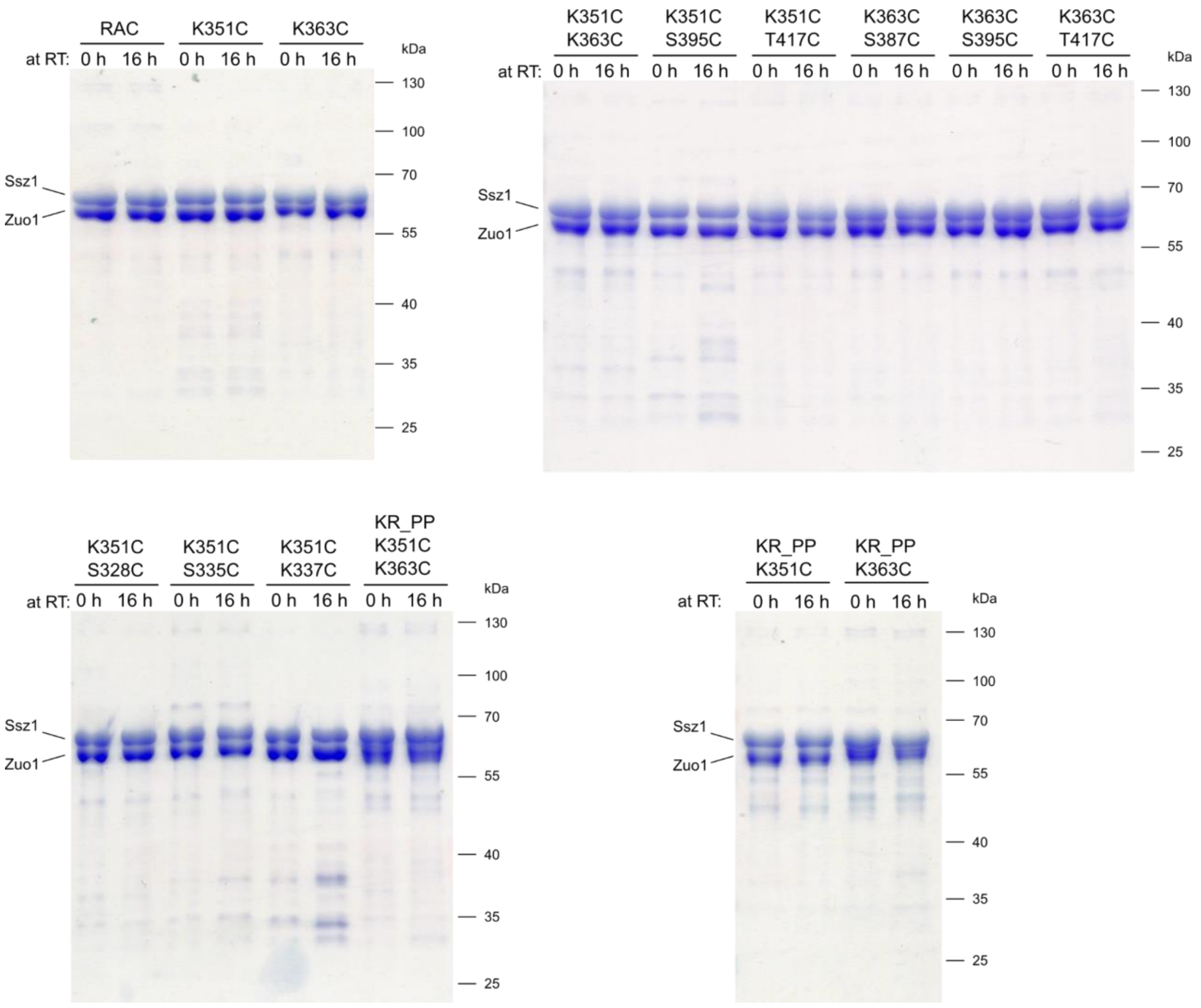

Fig. S3 Protein stability test with purified RAC variants. RAC was incubated for 0 h or 16 h at room temperature (RT) and loaded (2 µg) on SDS-PAGE. Shown are Coomassie Blue-stained gels. The two RAC variants (RAC K351C and K363C) used for RT CW EPR spectroscopy remained stable over time. For the double cysteine variants (measured by DEER in frozen state) RAC K351C S395C and RAC K351C K337C showed a slight degradation, indicated by the appearance of additional bands after 16 h at RT.

In vivo functionality test (Figure S4)

The *in vivo* function and integrity of the RAC variants were tested in yeast growth assays. All RAC variants were able to complement the known growth defect[3,6-8]. of the *RACΔ* (*ssz1Δzuo1Δ*) strain, even during translational stress caused by hygromycin B or L-Canavanine (Fig. S4).

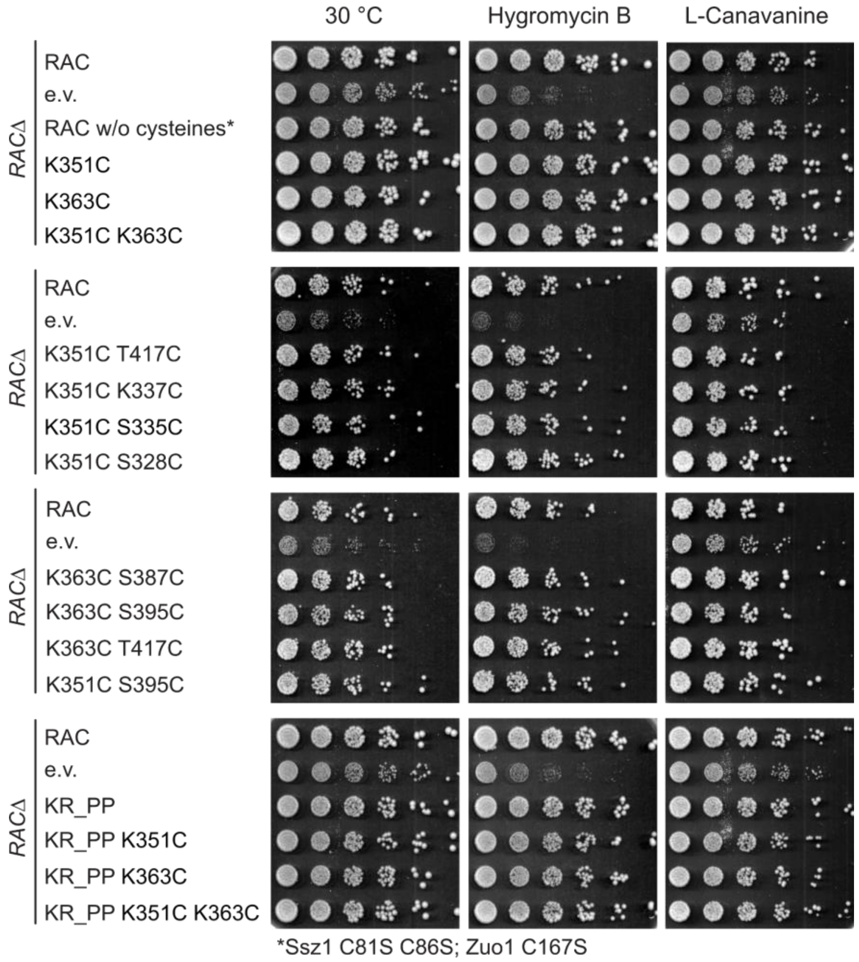

Fig. S4 Yeast growth assay to test *in vivo* functional integrity of RAC variants. Wt or *RAC*Δ (*ssz1*Δ*; zuo1*Δ) cells were transformed with either empty vectors (e.v.; negative control), vectors encoding for RAC (*SSZ1; ZUO1;* positive control) or for RAC variants (Table 1). Cultures were adjusted to the same OD_{600} and spotted on agar plates in fivefold serial dilutions. Indicated plates contained the translation inhibitory drugs hygromycin B or L-Canavanin. All plates were incubated at 30 °C for 2 or 3 days. Shown are representative plates from at least three biological replicates.

In vitro ribosome binding ability of RAC variants (Figure S5)

The ribosome binding ability of the RAC variants was tested in a co-sedimentation assay (Fig. S5). For the single cysteine RAC variants binding ability was not or only slightly affected (100-88% binding; Fig. S5a) but some combinations of two amino acid replacements resulted in a reduced binding e.g. for RAC K351C K337C, probably due to reduced charged in this variant. However, as EPR spectroscopy is a highly sensitive method that reports on all structural states we would expect to detect differences also in an inhomogeneous sample.

Single cysteine proline variants used for CW control experiments showed a strongly decreased ribosome binding (17% and 26%; Fig. S5A).
Mutant ribosomes lacking 10 terminal bases of ES12[1] also reduced the binding capacity of wt RAC and RAC variants(Fig. S5B).

a

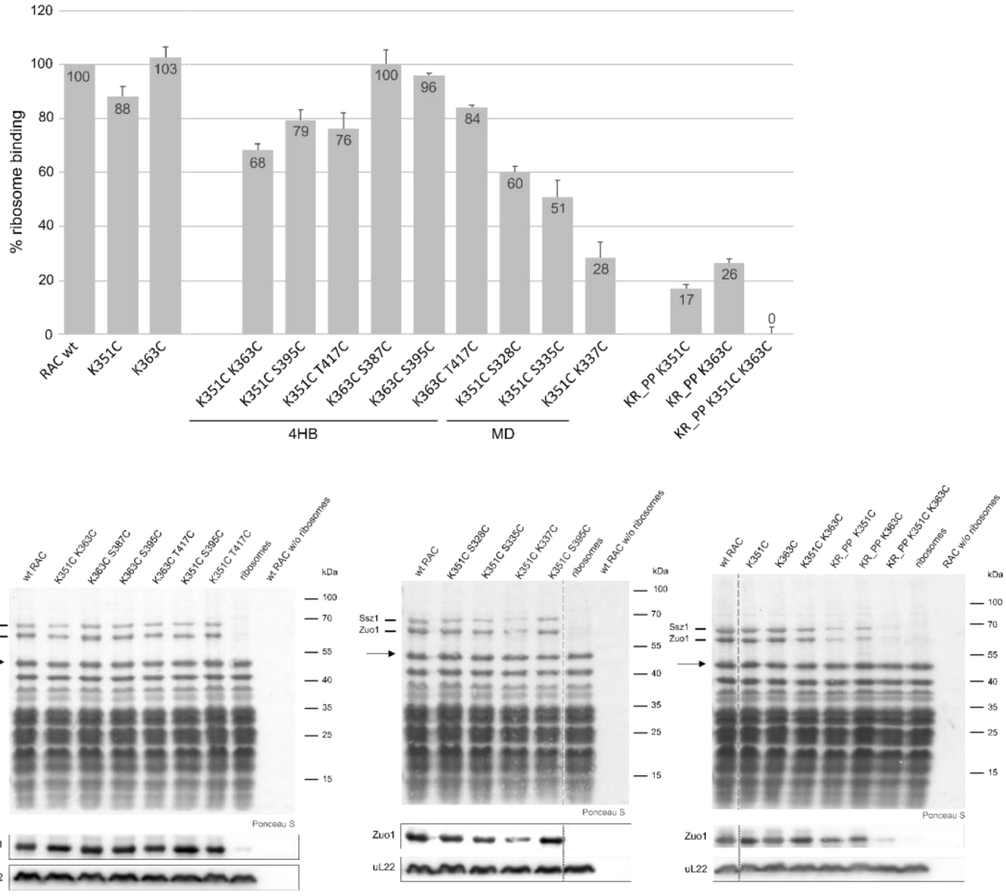

b

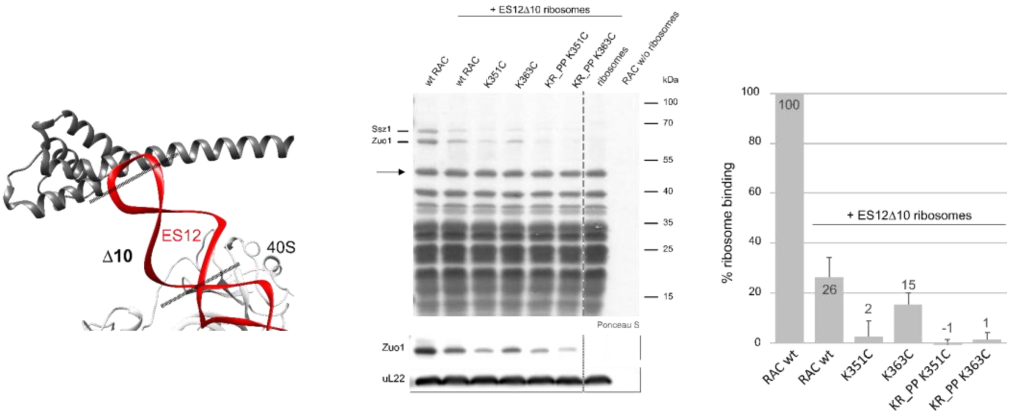

Fig. S5 ***In vitro*** **ribosome binding ability of RAC variants.** Shown are the quantification and original blots with (**a**) wt ribosomes or (**b**) ES12Δ10 ribosomes, lacking 10 terminal bases in ES12[1]. Purified RAC variants were mixed with 20% molar excess of yeast 80S ribosomes and incubated for 30 min at 30 °C. RAC-ribosome complexes were separated from unbound RAC by ultracentrifugation through a 25% (w/v) sucrose cushion. The ribosome-containing pellet was resuspended in buffer and samples analyzed in SDS-PAGE followed by western blotting. PonceauS-stained membrane was used for quantification. Signal intensity of RAC (Ssz1 & Zuo1) was set in relation to first ribosomal protein band (indicated by arrow head) below RAC (internal loading control) and corrected for background ratio (ribosomes sample). The ratio for wt RAC was set to 100%. Error bars represent s.e.m of at least three independent experiments. Presence of RAC was verified with antibody against Zuo1 and presence of ribosomes by antibody against ribosomal protein uL22.

DEER raw data corresponding to figure 4 (Figure S6)

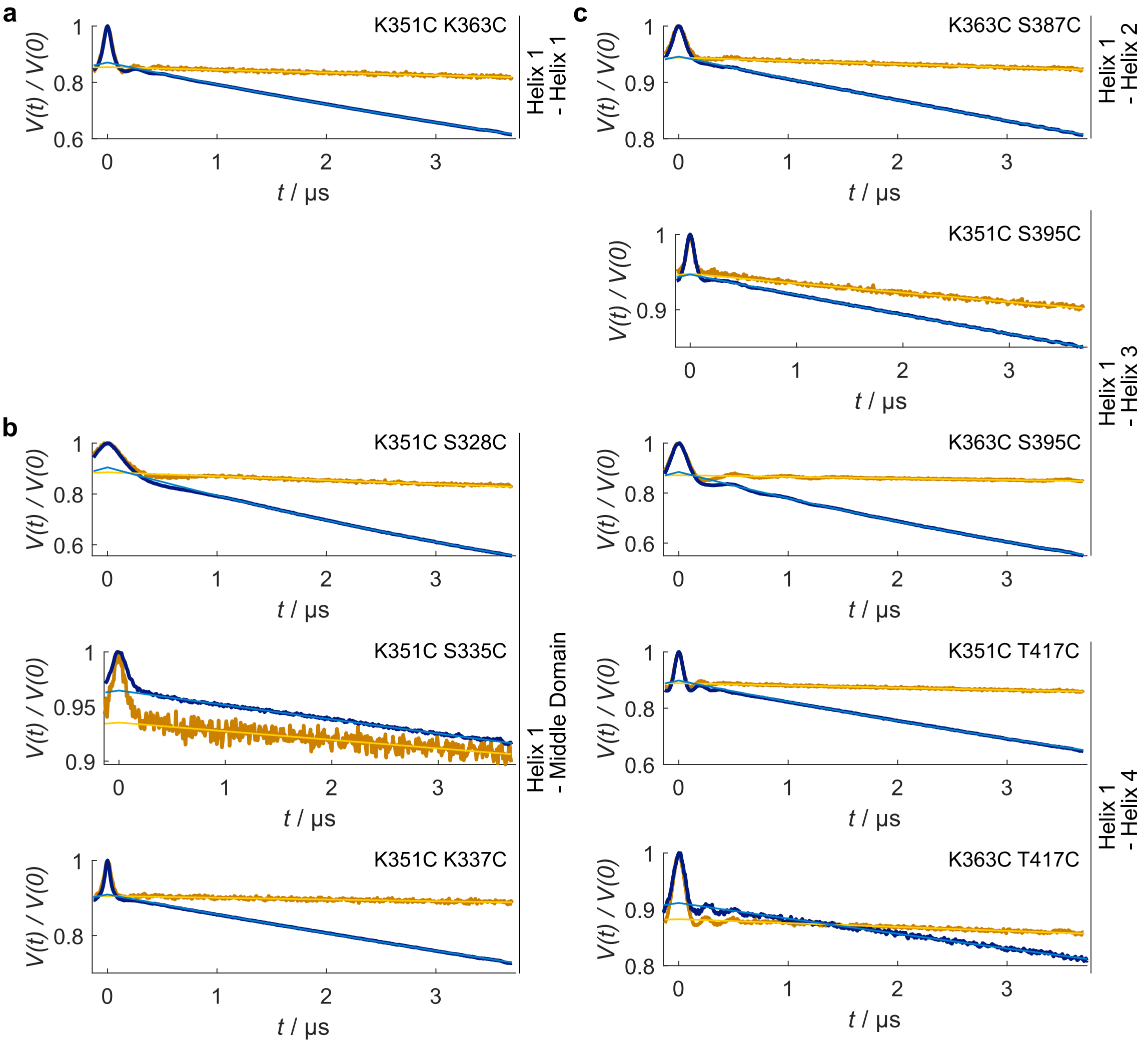

Fig. S6 DEER raw data for RAC with and w/o ribosomes corresponding to Fig. 4. DEER echo amplitudes *V(t) / B(t)* (light blue and light yellow) and corresponding background fits (dark blue and dark yellow) of Proxyl-labeled RAC variants in solution (blue) or complexed with ribosomes (yellow). RAC was labeled at (**a**) two sites within helix 1 of Zuo1's 4HB, (**b**) one site in helix 1 and a second site in helix 2-4, or (**c**) one site in helix 1 and a second site in the middle domain.

Form factors of RAC with vacant ribosomes (Figure S7)

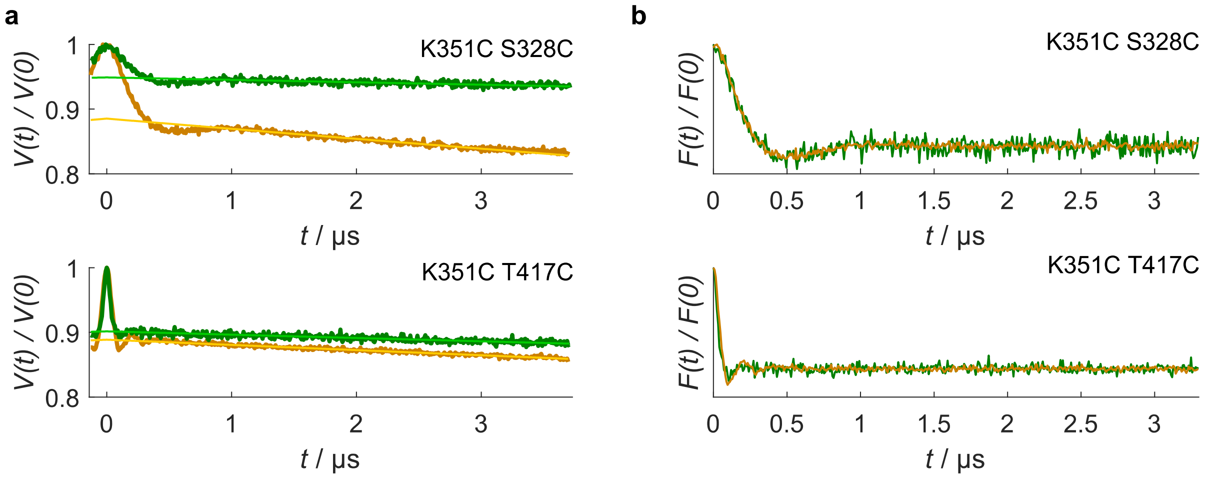

Fig. S7 DEER data for RAC incubated with either vacant (yellow) or presumably more active ribosomes (green). (**a**) DEER raw data (dark green and dark yellow) were background corrected with the use of neuronal network processing (DEERNet,[9] light green and light yellow) to reveal (**b**) form factors *F(t)/F(0)* which are similar for both ribosome types. In the standard procedure ribosomes were treated with puromycin to release polypeptide, whereas in an adapted protocol we aimed to purify ribosomes with nascent chain by the abandonment of puromycin and the subsequent collection of the actively translating polysome fraction. Compared are the measurements of RAC K351C S328C (helix 1–MD) and RAC K351C T417C (helix 1–helix 4 in 4HB).

Proline-induced unfolding (Figure S8)

In silico molecular dynamics simulations were used to reconstitute proline-induced unfolding in helix 1 of the 4HB. We tried different substitutions and finally determined K355 R359 as the most promising positions for proline substitutions. The RAC variant RAC K351C K355P R359P K363C (referred to as RAC KR_PP K351C K363C) showed a fast irreversible and robust unfolding of helix 1 in two independent 1000 ns simulations (Fig. S7A). To verify the unfolding experimentally, we determined the length between the flanking labeling sites K351C and K363C by DEER (Fig. S7b&c). An overlay of the form factors of Proxyl-labeled RAC K351C K363C and the respective proline variant (RAC KR_PP K351C K363C) displayed apparent differences in the oscillation pattern (Fig. S7B). Consistent with this, the obtained distance distributions varied between the two RAC variants (Fig. S7c).

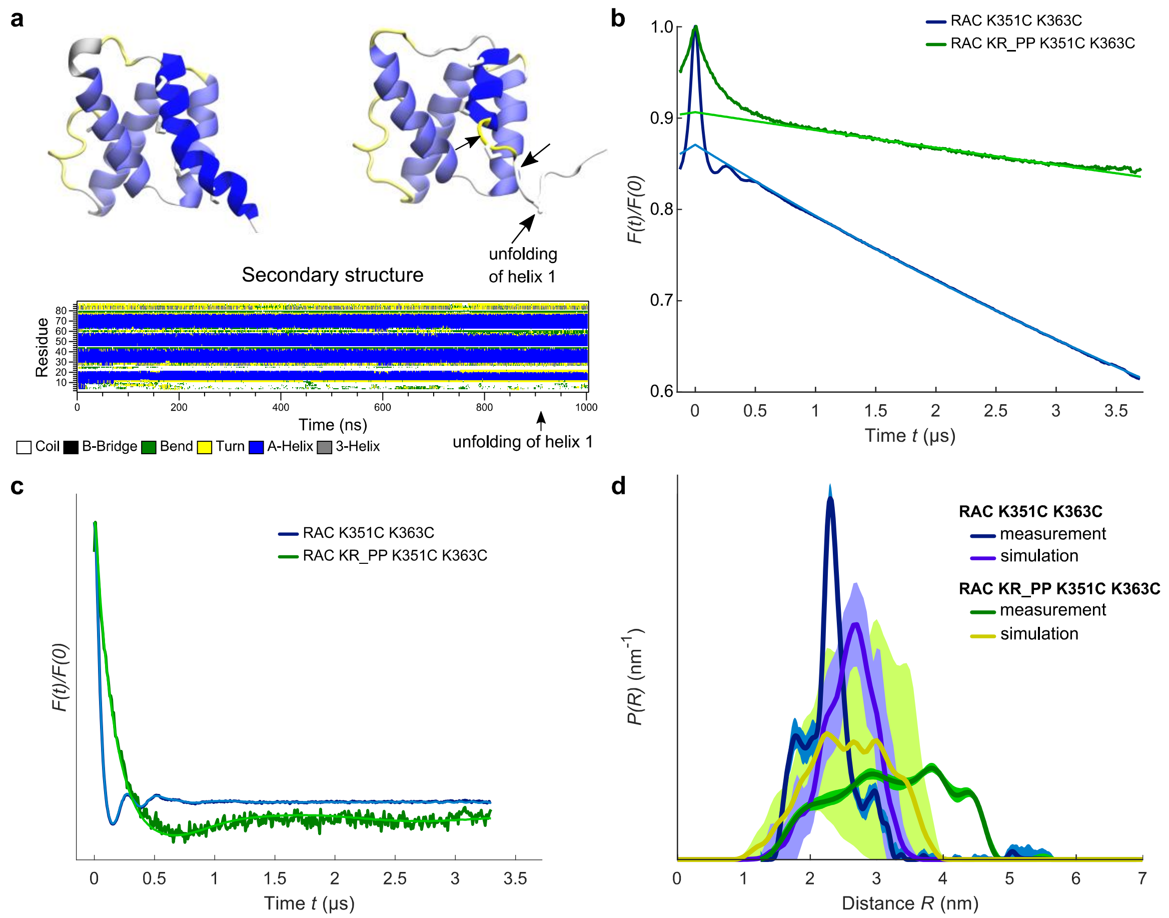

Fig. S8 Proline-induced unfolding of helix 1 in Zuo1's 4HB was proved by DEER distance measurements. (**a**) Simulation of unfolding of helix 1 in the RAC variant RAC KR_PP K351C K363C. Cartoon representation of the starting and end structures of the simulation with depicted cysteine and proline mutations. The lower panel shows the timeline of the secondary structure of the protein. During the course of simulation, helix 1 unfolds rapidly up to the second proline mutation (R359P). Similar behavior is observed in a second independent simulation (data not shown). (**b**) DEER raw data for Proxyl-labeled RAC K351C K363C (dark blue) and RAC KR_PP K351C K363C (dark green) including background fits obtained by neuronal network processing (DEERNet,[9] light blue and light green). (**c**) Background corrected form factors (dark blue and dark green) and corresponding Tikhonov regularization fits for Proxyl-labeled RAC K351C K363C (light blue) and RAC KR_PP K351C K363C (light green). (**d**) Distance distributions *P(R)* obtained from (**b**) by Tikhonov regularization and corresponding simulations (light blue for RAC K351C K363C, light green for RAC KR_PP K351C K363C). Shaded areas represent uncertainties of the experimentally obtained distance distribution derived from data post-processing using an ensemble of reconstructed background models from DEERNet[9] or standard deviation of clustered structures from two independent MD simulations.

C. REFERENCES

1 Lee, K., Sharma, R., Shrestha, O. K., Bingman, C. A. & Craig, E. A. Dual interaction of the Hsp70 J-protein cochaperone Zuotin with the 40S and 60S ribosomal subunits. *Nat. Struct. Mol. Biol.*, doi:10.1038/nsmb.3299 (2016).

2 Sikorski, R. S. & Hieter, P. A system of shuttle vectors and yeast host strains designed for efficient manipulation of DNA in Saccharomyces cerevisiae. *Genetics* **122**, 19-27 (1989).

3 Yan, W. *et al.* Zuotin, a ribosome-associated DnaJ molecular chaperone. *EMBO J.* **17**, 4809-4817, doi:10.1093/emboj/17.16.4809 (1998).

4 Svidritskiy, E., Brilot, A. F., San Koh, C., Grigorieff, N. & Korostelev, A. A. Structures of yeast 80S ribosome-tRNA complexes in the rotated and nonrotated conformations. *Structure* **22**, 1210-1218, doi:10.1016/j.str.2014.06.003 (2014).

5 Ducett, J. K. *et al.* Unfolding of the C-terminal domain of the J-protein Zuo1 releases autoinhibition and activates Pdr1-dependent transcription. *J. Mol. Biol.* **425**, 19-31, doi:10.1016/j.jmb.2012.09.020 (2013).

6 Gautschi, M. *et al.* RAC, a stable ribosome-associated complex in yeast formed by the DnaK-DnaJ homologs Ssz1p and zuotin. *Proc. Natl. Acad. Sci. U. S. A.* **98**, 3762-3767, doi:10.1073/pnas.071057198 (2001).

7 Kim, S. Y. & Craig, E. A. Broad sensitivity of Saccharomyces cerevisiae lacking ribosome-associated chaperone ssb or zuo1 to cations, including aminoglycosides. *Eukaryot. Cell* **4**, 82-89, doi:10.1128/EC.4.1.82-89.2005 (2005).

8 Michimoto, T., Aoki, T., Toh-e, A. & Kikuchi, Y. Yeast Pdr13p and Zuo1p molecular chaperones are new functional Hsp70 and Hsp40 partners. *Gene* **257**, 131-137 (2000).

9 Worswick, S. G., Spencer, J. A., Jeschke, G. & Kuprov, I. Deep neural network processing of DEER data. *Sci Adv* **4**, eaat5218, doi:10.1126/sciadv.aat5218 (2018).
